# The retinal toxicity profile towards assemblies of Amyloid-β indicate the predominant pathophysiological activity of oligomeric species

**DOI:** 10.1038/s41598-020-77712-9

**Published:** 2020-12-01

**Authors:** Efrat Naaman, Sarah Ya’ari, Chen Itzkovich, Shadi Safuri, Flora Macsi, Lior Kellerman, Michael Mimouni, Irit Mann, Ehud Gazit, Lihi Adler-Abramovich, Shiri Zayit-Soudry

**Affiliations:** 1 Department of Ophthalmology, Rambam Health Care Campus, 3109601 Haifa, Israel; 2 Department of Oral Biology, The Goldschleger School of Dental Medicine, Sackler Faculty of Medicine, Tel Aviv University, 69978 Tel Aviv, Israel; 3 Clinical Research Institute at Rambam, Rambam Health Care Campus, 3109601 Haifa, Israel; 4 Ruth and Bruce Faculty of Medicine, Technion Israel Institute of Technology, Haifa, Israel; 5 School of Molecular Cell Biology and Biotechnology, Tel Aviv University, 69978 Tel Aviv, Israel

**Keywords:** Protein aggregation, Molecular medicine, Retina

## Abstract

Amyloid-β (Aβ), reported as a significant constituent of drusen, was implicated in the pathophysiology of age-related macular degeneration (AMD), yet the identity of the major pathogenic Aβ species in the retina has remained hitherto unclear. Here, we examined the *in-vivo* retinal impact of distinct supramolecular assemblies of Aβ. Fibrillar (Aβ40, Aβ42) and oligomeric (Aβ42) preparations showed clear biophysical hallmarks of amyloid assemblies. Measures of retinal structure and function were studied longitudinally following intravitreal administration of the various Aβ assemblies in rats. Electroretinography (ERG) delineated differential retinal neurotoxicity of Aβ species. Oligomeric Aβ42 inflicted the major toxic effect, exerting diminished ERG responses through 30 days post injection. A lesser degree of retinal dysfunction was noted following treatment with fibrillar Aβ42, whereas no retinal compromise was recorded in response to Aβ40 fibrils. The toxic effect of Aβ42 architectures was further reflected by retinal glial response. Fluorescence labelling of Aβ42 species was used to detect their accumulation into the retinal tissue. These results provide conceptual evidence of the differential toxicity of particular Aβ species *in-vivo*, and promote the mechanistic understanding of their retinal pathogenicity. Stratifying the impact of pathological Aβ aggregation in the retina may merit further investigation to decipher the pathophysiological relevance of processes of molecular self-assembly in retinal disorders.

## Introduction

With a patient population exceeding 170 million people worldwide, age-related macular degeneration (AMD) is the leading cause of vision loss in patients aged 65 years or older in developed countries^1^. The global burden of AMD is estimated to rise further with the increasing life expectancy of the population, with nearly half of individuals older than 75 years exhibiting this condition^2^. Drusen, deposits of extra-cellular material accumulating under the retinal pigment epithelium (RPE) cells in the macular center, are the clinical hallmark of the disease. Although considered among the earliest manifestations of AMD, drusen may be associated with disruption and thinning of the overlying RPE and photoreceptors^3–5^ and can affect the visual function even in the absence of advanced disease^6,7^. Despite intensive basic and clinical research, stratifying the mechanisms of progression and causes of vision loss remains a challenge, and no disease-modifying treatment is presently available to prevent the continuous cellular damage of the neurosensory retina or RPE in eyes with AMD. Research efforts have focused on delineating the ultrastructure and composition of drusen in pursuit of insights into their relation to the disease process. Amyloid-β (Aβ), a group of aggregation-prone polypeptides associated with the brain pathology in Alzheimer’s disease, have been localized in drusen and have thus been implicated in the pathophysiology of AMD^8–10^. Aβ is derived from the sequential enzymatic proteolysis of the ubiquitous amyloid precursor protein (APP) resulting in the formation of soluble Aβ monomers containing 39–43 amino acids, with Aβ40 and Aβ42 being the two most common isoforms^11^. Aβ monomers spontaneously aggregate into various types of assemblies, including dimers, trimers and oligomers, whereas additional pathways of self-assembly can lead to higher ordered deposits such as protofibrils and fibrils, ultimately forming amyloid plaques^12^. Excess Aβ is suggested to be a key factor in neurodegeneration and impairment of structure and function due to the inherent toxicity of aggregated species^13–18^. Central nervous system (CNS) models showed that soluble oligomers are the primary neurotoxic species^19,20^, and overload of soluble Aβ was shown as a determinant of the severity of neurodegeneration in Alzheimer’s disease^12,21^.

In the retina, Aβ was shown to originate from the RPE, inner nuclear layer ^22–26^ and retinal ganglion cells before accumulating in the vitreous^27–29^. Increasing experimental evidence demonstrates a clear association between Aβ deposition and compromised retinal integrity through direct cytotoxic effects ^30,31^, exertion of mitochondrial dysfunction and oxidative stress ^32^, as well as promotion of chronic inflammation^33,34^. It is postulated that Aβ has the capacity to trigger diverse pathogenic pathways in the retina, similar to the pathogenic events occurring in the CNS^15,35^. Nonetheless, whereas ample evidence supports the key role of soluble oligomeric Aβ in neurodegenerative brain disorders, the major pathogenic species exerting toxic effects in the retina is yet to be clearly defined. Presently, the relationship between particular Aβ assemblies, degeneration of the neuroretina, and impairment of retinal function are not yet well understood.

Here, we aimed to study the impact of distinct supramolecular Aβ assemblies on the retina *in-vivo,* in order to ascribe the toxic Aβ effect to structures reportedly present in drusen. Experimental rats were treated with well-defined Aβ nanostructures, including soluble oligomeric Aβ42 and fibrillary preparations of Aβ42 and Aβ40, administered via intravitreal injection. Retinal structure and function were subsequently measured by electroretinography (ERG) and retinal immunohistochemistry. We found that oligomeric Aβ42 is the principal toxic entity in the retina, while fibrillar Aβ42 mediated a lesser degree of retinal damage. In contrast, Aβ40 fibrils exerted no harmful effects on the retinal function. The toxic impact of Aβ42 organizations was further reflected by retinal glial response in treated eyes, evident by positive glial fibrillary acidic protein (GFAP) labelling. Fluorescence labelling of Aβ species^36^ was used to follow their accumulation into the retinal tissue.

This is the first report stratifying the nature of Aβ assemblies possessing retinal toxicity in vivo. The results of this study provide conceptual and mechanistic insights on the pathophysiology of vision loss associated with amyloid-related retinal disease, and may support the future development of novel treatment approaches to target associated vision loss.

## Results

### Formation and biophysical characterization of ordered Aβ assemblies in vitro

**Figure 1:**
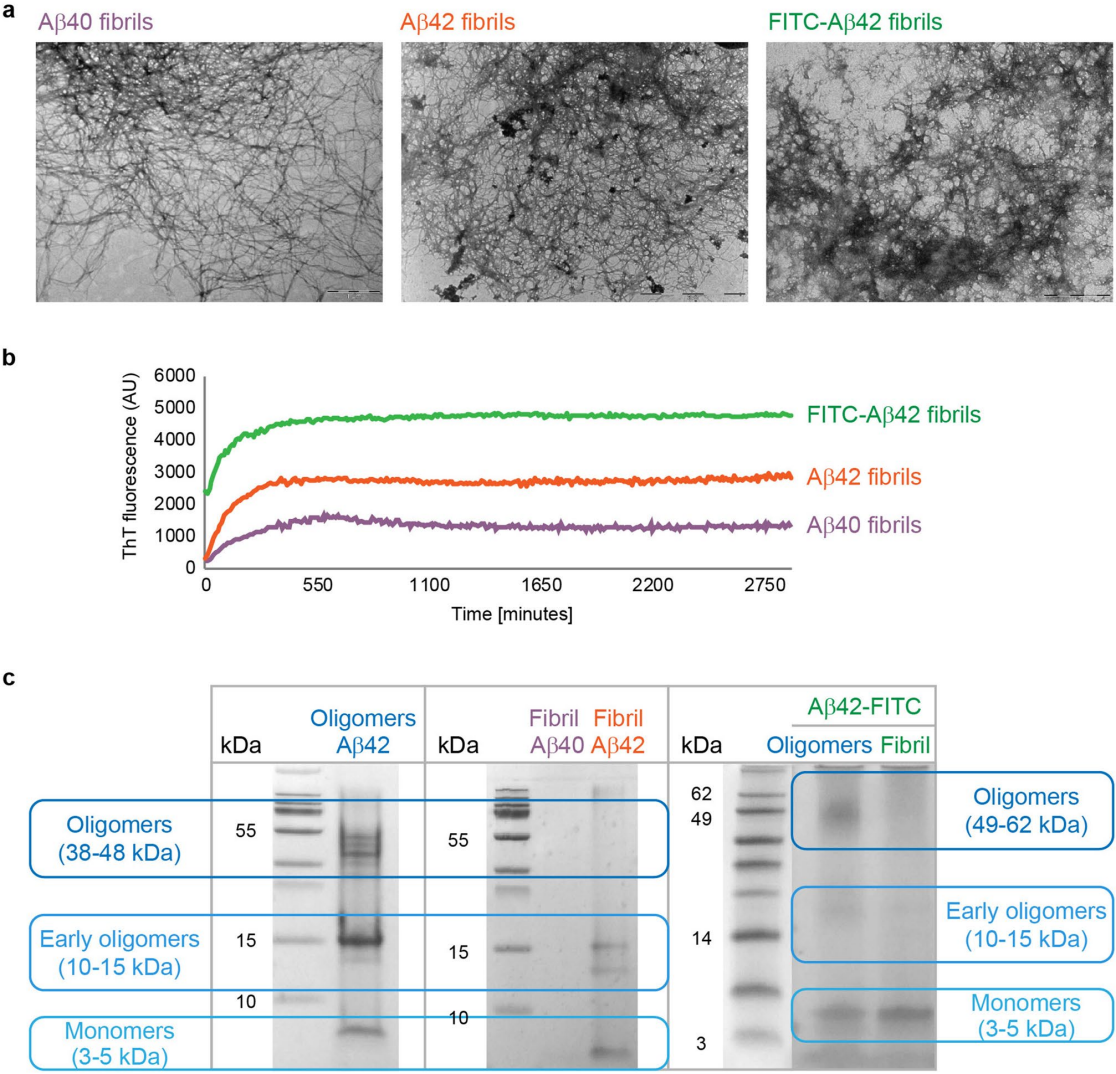
Aβ42, FITC-Aβ42 and Aβ40 self-assemble into fibrillary and oligomeric structures. (**a**) TEM images of Aβ40, Aβ42 and FITC-Aβ42, respectively, all showing typical structure of elongated fibrils. Scale bar: 1 µm. (**b**) ThT kinetic analysis of Aβ40, Aβ42 and FITC-Aβ42 fibril formation. (**c**) Gel electrophoresis for (left) oligomeric Aβ42, (middle) fibrillar Aβ40 and Aβ42, and (right) oligomeric and fibrillar FITC-Aβ42 assemblies. Full-length gels are presented in Supplementary Figure S1.

In order to comparatively portray the retinal effect of distinct supramolecular Aβ aggregates, we formed oligomeric Aβ42 and fibrillar assemblies of Aβ40 and Aβ42. The method we employed for synthesis of oligomers is based on the approach described by Hillen and colleagues, which enables the reproducible generation of homogenous and stable oligomeric globular assemblies^37^, which are highly unlikely to convert into fibrillary assemblies. The application of this specific method allows to distinguish between the effects of the various supramolecular organizations. In addition we also formed fluorescent oligomeric as well as fibrillary assemblies, using Aβ42 labelled with Fluorescein isothiocyanate (FITC). A combination of methodologies was employed to characterize the biophysical properties of the derived Aβ supramolecular structures and to examine whether they possess the hallmarks of amyloid assemblies. Transmission electron microscopy (TEM) analysis of the Aβ40, Aβ42 and FITC-Aβ42 fibrils preparations confirmed the presence of ordered structures presenting an elongated morphology with a diameter of about 7–10 nm, forming dense fibrillar networks in vitro (Fig. 1a). Furthermore, we used the thioflavin-T (ThT) binding assay to provide quantitative information on amyloid fibril growth, which demonstrated the enhanced emission with the typical kinetics of fibril formation (Fig. 1b).

Electrophoresis methods further substantiated the aggregation phases of the assemblies. Indeed, the oligomeric structures yielded distinct bands at the 35–55 kDa range coinciding with the oligomeric state of Aβ^37,38^, confirming the predominance of oligomers in the sample (Fig. 1c left, right columns). In contrast, the fibrillary structures showed no bands in the oligomeric range^39^, confirming fibrils predominance and the absence of soluble oligomeric intermediates in the solution (Fig. 1c middle, right columns). Thus, the synthesized Aβ fibrillar assemblies are characterized by a high degree of structural order, and typical amyloid properties.

### Impact of distinct Aβ assemblies on the retinal function in-vivo

**Figure 2:**
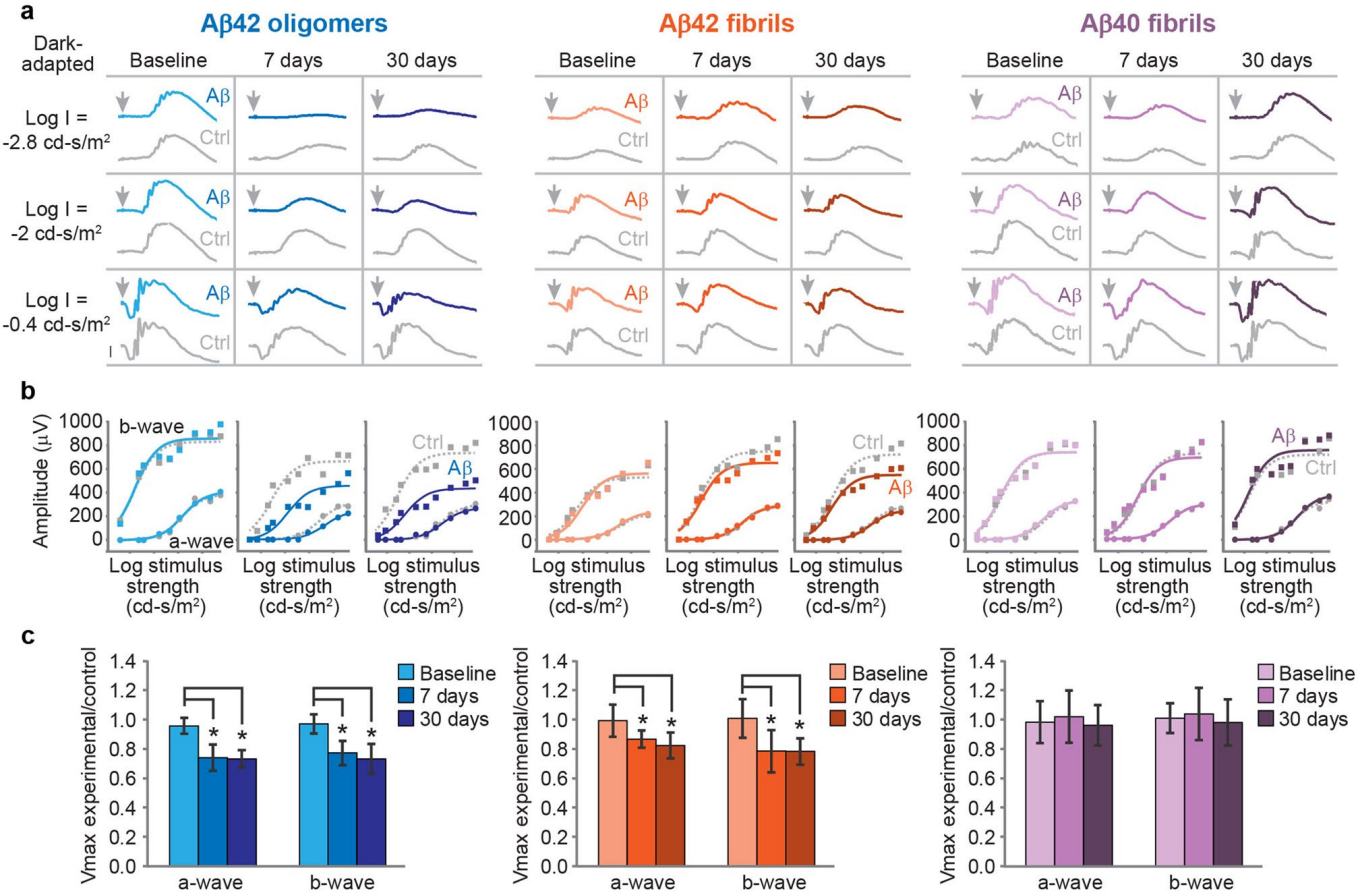
The effects of intravitreal Aβ on the electroretinographic (ERG) responses of the rat. (**a**, **b**) Present data from a single rat in each subgroup, (**c**) presents pooled data from all biological replicates. (**a**, left) **Aβ42 oligomers** diminish ERG amplitudes at both 7 and 30 days after injection. Dark-adapted ERG responses elicited by flashes of different strengths are shown for each recording session. The time of light stimulus is denoted by an arrow. Calibration bar: 200 µV. (**b**, left) Response-stimulus strength relationships for the dark-adapted ERG of the same rat. The curves were fitted to a hyperbolic type function. (**c**, left) Dependence of Aβ-induced impairment of the dark-adapted ERG responses upon time. Mean dark-adapted Vmax ratio of the a-wave and b-wave (n = 9 rats) showed a gradual decrease at each time point after the injection, indicating impaired retinal function by Aβ42 oligomers. A similar analysis is shown for rats treated with **Aβ42 fibrils** (n = 9) (**a**, **b**, **c** middle) and **Aβ40 fibrils** (n = 9) (a, b, c right), respectively. Aβ42 fibrils mediate mild compromise of the b-wave ERG amplitude, whereas Aβ40 fibrils do not impact the ERG.

To delineate the retinal toxicity of specific supramolecular Aβ entities, we examined their impact on retinal function *in-vivo*. Wild-type rats were treated with a particular species of Aβ, namely fibrillar Aβ40 (43 µM), Aβ42 (42 µM), or oligomeric Aβ42 (134 µM), administered via intravitreal injection into the right eye, whereas the left eye was treated with the vehicle only and served as control. For assessment of the effect on retinal function, ERG was performed at baseline, and 7 and 30 days after the injection (Fig. 2a). All rats treated with oligomeric Aβ42 exhibited deficient retinal function in the treated eye as early as 7 days after the injection, evident by diminished ERG amplitudes compared to the control eye (Fig. 2a, left panel). To quantify the impact of the Aβ entities on the retinal function we compared ERG a-waves and b-waves, representing photoreceptoral response and post-photoreceptoral response respectively. The ratio between the ERG a-wave and b-wave maximal amplitudes (Vmax) of the experimental versus the control eye was calculated in each rat. A significant retinal dysfunction was noted in treated eyes 7 days after the injection, with diminished a-wave and b-wave Vmax values compared to the control eye (Fig. 2b, left panel). The compromise in retinal function in the experimental eye persisted through 30 days after the injection.

Rats treated with fibrillary Aβ42 displayed a milder decrease of ERG responses in the experimental eyes at 7 and 30 days after the injection (Fig. 2a, middle panel), and consistently, the derived a-wave and b-wave Vmax values showed merely mild deficit at both follow-up time points (Fig. 2b, middle panel). In contrast, in rats treated with fibrils of Aβ40 no significant difference between the ERG responses from the experimental and control eyes was noted at 7 and 30 days after the injection (Fig. 2a, right panel), and consistently, the a-wave and b-wave Vmax values obtained from the experimental and control eyes were similar at both follow-up time points (Fig. 2b, right panel).

A similar approach was undertaken for all experimental subgroups, whereas the mean ratio between the Vmax of the experimental and control eyes calculated at each recording session was used to assess the degree of functional damage to the neurosensory retina in response to each Aβ species (Fig. 2c). At baseline, the ERG responses were similar between the experimental and control eyes in all study groups, and the Vmax ratio values were accordingly equivalent. Notably, in rats treated with oligomeric Aβ42 a significant reduction of the mean a-wave and b-wave Vmax values from the experimental eyes compared to the control eyes was noted 7 and 30 days after the injection (p < 0.05 for all) and the derived ratios range was accordingly reduced to 0.73–0.77, indicating a detrimental effect on the retinal function (Fig. 2c, left panel). Rats treated with fibrillar Aβ42 showed a milder deficit in the ERG responses, with a greater impact on the b-wave Vmax amplitudes (range 0.78–0.79) than the a-wave Vmax amplitudes (range 0.82–0.87) (p < 0.05 for all), (Fig. 2C, middle panel). In the experimental group treated with Aβ40 fibrils (Fig. 2c, right panel), the a-wave and b-wave Vmax ratios remained roughly equivalent (range 0.96–1.07), indicating no significant deleterious effect on the retinal function.

### Analysis of abnormal ERG components

**Figure 3:**
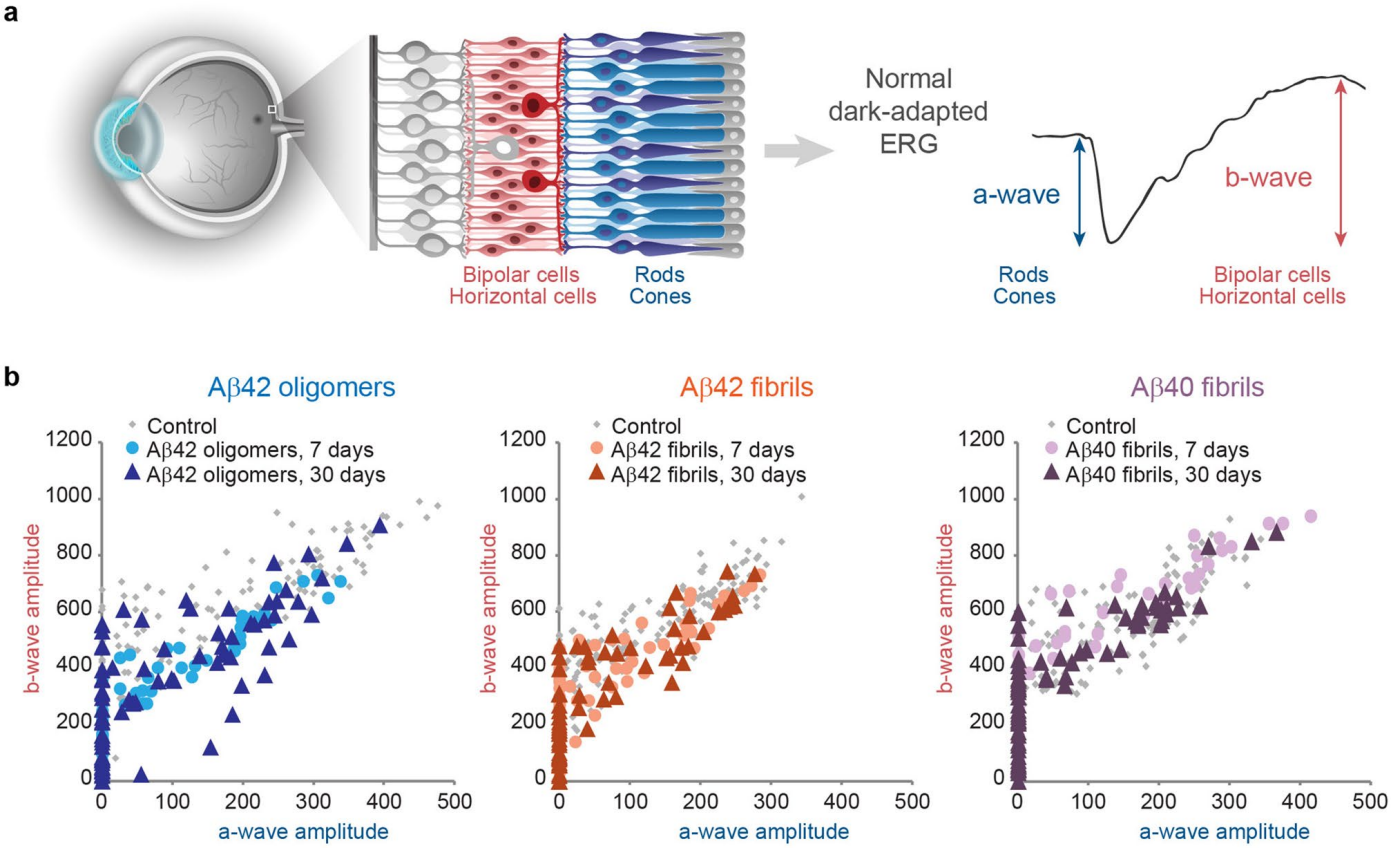
(**a**) The a-wave and the b-wave of the dark-adapted electroretinogram represent the functional status of the retinal photoreceptors and of their second-order neurons, including bipolar and horizontal cells, respectively. (**b**) The relationship between the amplitudes of the b-wave and the a-wave obtained from experimental eyes treated with oligomeric Aβ42 (left panel), fibrillar Aβ42 (middle panel) and fibrillar Aβ40 (right panel) are illustrated for each recording session after the injection.

To quantitatively assess the deviation in the ERG pattern, the relationship between the amplitude of the a-wave and the b-wave was determined for each response^40^. Fig. 3 depicts the normal range of measures obtained from the control rats’ eyes and provides an indication on the mechanism of ERG configuration (Fig. 3a). In a normal eye there is a constant relationship between the waves, and damage to the photoreceptors would result in a proportional decrease in the post-photoreceptoral response. In the case of aberrant dependence between the photoreceptoral response and post-photoreceptoral response resulting from retinal toxicity affecting different subcomponents of the tissue, a deviated relationship from the normal range is expected.

The ERG measures obtained from the experimental eyes 7 days after injection of oligomeric Aβ42 were characterized by subnormal amplitudes, with the dependence of the b-wave on the a-wave remaining within the normal range of correlation (Fig. 3b, left panel), indicating that the deficient ERG amplitudes likely resulted mostly from subnormal photoreceptor response, with largely intact signal transmission in the retina. Interestingly, 30 days after the injection, the plot of the b-wave versus the a-wave showed marginally low values. These measures suggest that signal transmission in the retina became impaired over time in response to oligomeric Aβ42, resulting in a measured b-wave amplitude that is smaller than expected for the corresponding a-wave amplitude. In rats treated with either fibrillar Aβ42 (Fig. 3b, middle panel) or Aβ40 (Fig. 3b, right panel), the b-to-a ratio remained within the normal range, indicating intact retinal signal transmission throughout the 30 days of follow-up in each experimental group.

### Localization and dynamics of the toxic Aβ assemblies in the retina

**Figure 4:**
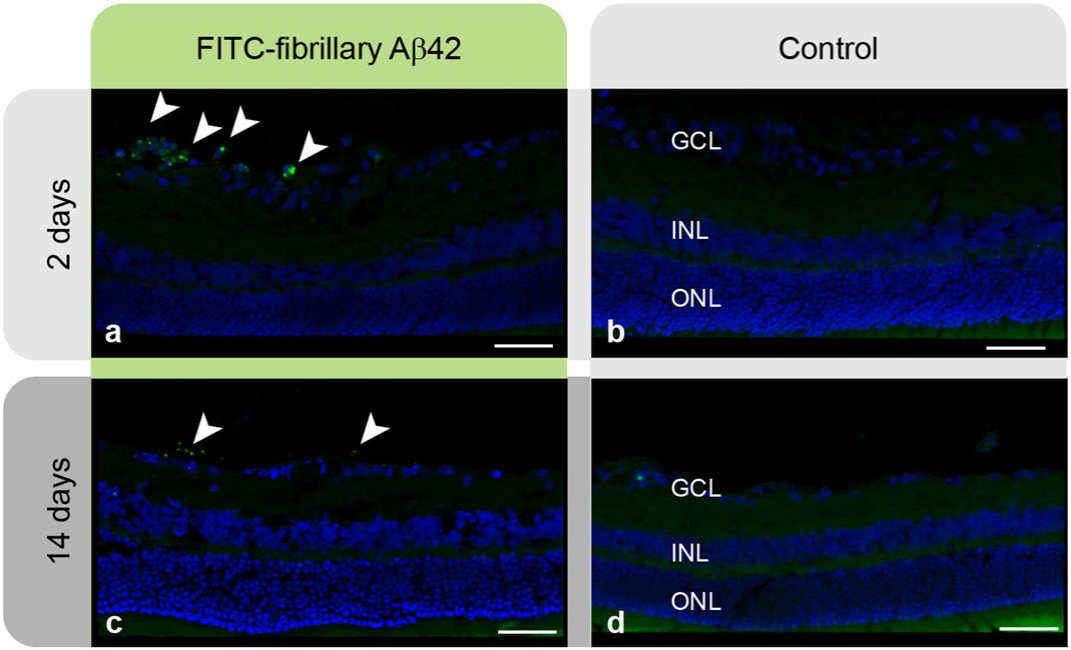
Localization of injected FITC-Aβ42. (**a**) Retinal section from an experimental eye treated with FITC-labelled Aβ42 fibrils (green fluorescence) 2 days after injection. (**b**) Corresponding retinal section from the control eye. (**c**) Signal noted 14 days after the injection in the experimental eye, but not in the control eye (**d**). Results were replicated in six rats in each subgroup. Scale bars: 40 µm.

Fluorescein isothiocyanate (FITC)-conjugated Aβ42 was employed to localize and track penetrance of oligomeric and fibrillar assemblies from the vitreous compartment into the retina. Retinal sections from rats eyes treated with intravitreal administration of FITC-Aβ42 fibrils confirmed the localization of fluoresce-labelled structures in the inner retina 2 days after injection (Fig. 4a, b). Focal clusters of discrete green fluorescence were seen along consecutive sections from various retinal locations. Smaller clusters of the fluorescence-labelled signal were still noted along the inner retinal layers in treated eyes 14 days post injection (Fig. 4c, d).

### Impact of distinct Aβ assemblies on the retinal morphology in-vivo

To determine whether acute exposure to Aβ can adversely affect the retinal structure, we performed morphological assessment. Retinal histologic examination using Richardson’s blue staining did not reveal major morphologic changes in the retinal layer architecture. We did not identify reduction in the average thickness of the outer nuclear layer (ONL) or the entire neurosensory retina in eyes treated with oligomeric Aβ42 in comparison to the controls (Figure S2). This observation was repeated in all other treated eyes (results not shown).

**Figure 5:**
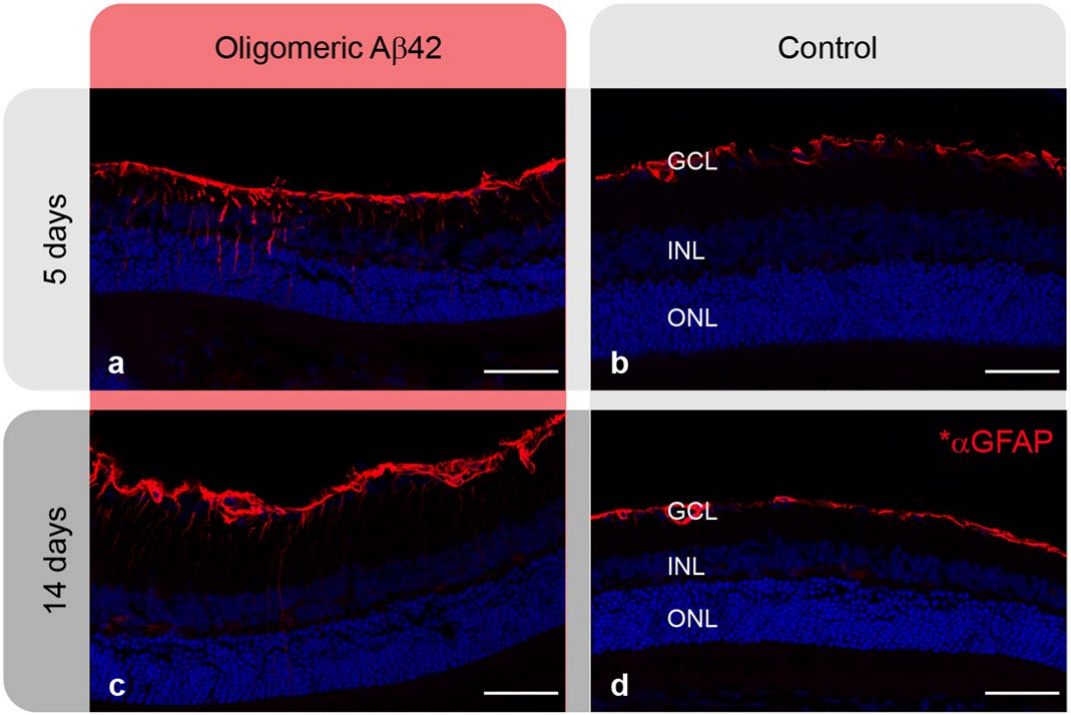
Immunostaining for glial fibrillary acidic protein (GFAP) in retinas of a rat treated with intravitreal oligomeric Aβ42. Retinal sections from the peripheral retina in the experimental eyes (**a**, **c**) show significant GFAP staining of cells with a typical morphology for Müller cells. (**b**, **d**) Peripheral retinal areas from the control eyes demonstrate no labelling of Müller cells. (Red- GFAP, Blue- DAPI staining of all nuclei). To obtain this data 16-μm tissue sections at five different tissue planes (100 μm apart) were prepared and stained. Results were replicated in four rats in each group. Scale bars: 50 µm.

Immunoreactivity for glial fibrillary acidic protein (GFAP) was assessed in the retinas of the experimental and control eyes of all rats. GFAP is regularly expressed in retinal astrocytes, but not in Müller cells. However, in response to retinal injury or disease causing Müller glial cells activation, GFAP immunoreactivity is expressed also in these cells and is a well-known marker for retinal stress. Figure 5 shows micrographs of retinal sections from the experimental and control eyes of rats treated with Aβ42 oligomers. The peripheral retinal section of experimental eyes exhibits intense GFAP immunoreactivity in radial structures that are typical for retinal Müller cells (Fig. 5a, c), whereas no such staining is seen in the peripheral retina of the control eyes (Fig. 5b, d). Similar findings were seen in all rats from the Aβ42 oligomers group and Aβ42 fibrils group (Figure S3).

## Discussion

Recent clinical and experimental evidence associated the aggregation-prone Aβ peptides with the pathogenesis of AMD^41–44^. Ultrastructural studies identified the presence of fibrillary and oligomeric assemblies within drusen in eyes with AMD, and the frequency and extent of Aβ deposition was linked with the formation and retinal disease progression^10,22,45^. Whereas Aβ is thought to be a potential factor in the progression of AMD^46–48^, to date, the major pathogenic species in the retina have not been conclusively determined.

Our study provides the first direct evidence of the differential retinal toxic profile of Aβ species, which supports previous observations. We also demonstrate the effect of Aβ species on the functional integrity of the retina *in-vivo*. Our results show that rats treated with distinct Aβ assemblies administered via intravitreal injection displayed a varying level of retinal dysfunction, with the oligomeric form of Aβ42 exerting the predominant retino-toxic effect. In response to oligomeric structures of Aβ42, a significant decrease in ERG a-wave and b-wave amplitudes was noted seven days post injection, highlighting photoreceptoral compromise, with an additional deleterious effect on neuronal pathways of post-photoreceptoral signal transduction in the retina. After 30 days the ERG configuration, consisting of diminished correlation between the b-wave amplitudes with respect to the a-wave amplitudes, indicated post-receptor neouroretinal damage affecting ON-bipolar pathways in eyes injected with oligomeric Aβ42. The toxic impact was further evident by activation of glial response in the retina. The toxic impact persisted through 30 days, and no functional recovery was observed. Histologic findings did not show retinal thinning even in the photoreceptor nuclear cell layer, presumably due to the maximal follow-up duration of 5 weeks. Data on the effect of Aβ application on measures of retinal thickness and retinal cell loss in vivo varies in the literature^49^. A milder toxic effect was noted in eyes injected with fibrillary assemblies of Aβ42, characterized by smaller diminution of the ERG a-wave and b-wave amplitudes, and less prominent activation of glial response. In contrast, no detrimental effect was noted following exposure to fibrils of Aβ40, and the ERG responses in these eyes were intact.

The acute-exposure model employed in this study provides a useful and relatively simple experimental system, which enabled the characterization of the biophysical state of the Aβ assemblies prior to injection. It is hypothesized that under physiological conditions, the retina is constantly exposed to Aβ, which is secreted at basal state in a continuous manner^28,29,45^. However, in contrast to the soluble monomeric state in which the peptide is naturally released, certain supramolecular architectures formed by spontaneous self-assembly of the monomers are known to possess cytotoxic properties^11–15^. Our results provide *in-vivo* evidence for the variance of retino-toxic profile carried by the different assemblies, utilizing a method of direct intraocular delivery which enables the generation of a physiologically relevant model that reflects the specific impact of each Aβ species. Consistent with previous data obtained in CNS models^11,13,15,17,19^, our study underscores the oligomeric species as the major toxic entity of Aβ in the retina.

We found a prominent discrepancy between the retino-toxic impact of assemblies of Aβ42, and fibrillary aggregates of the Aβ40 isoform, which caused no apparent retinal dysfunction through 30 days of follow-up. Aβ40 was reported as the prevalent isoform in human cerebrospinal fluid and plasma, as well as in the eye and drusen^10,28,50^. It was shown to promote immune responses and activate inflammatory pathways in RPE cells^34,51,52^, although others reported on comparable toxic effects of Aβ40 and Aβ42 on human cell lines^53^. In contrast, several studies have pointed to Aβ42 as the key factor in neurotoxicity and impairment of synaptic transmission in Alzheimer’s disease^54^, and this isoform has been shown to inflict elevated levels of intracellular calcium, oxidative stress, and receptor-mediated activation of programmed cell death^20,32,55^. Our results support the predominant toxic profile of Aβ42 in the retina *in-vivo,* and support the hypothesis that this highly-aggregative peptide may contribute to retinal cell damage in AMD. Nonetheless, the mechanism of pathological secretion and aggregation of Aβ42 in eyes with AMD presently remains poorly understood.

We used FITC labelling to track the incorporation of the investigated Aβ molecules from the vitreous compartment into the retina. In retinal sections from eyes injected with FITC-labelled oligomeric Aβ42, no evident fluorescence was noted. As oligomeric assemblies are typically too small to be identified by optical microscopy, this observation suggests that while the injected oligomers are capable of inflicting global retinal dysfunction soon after the injection, these small soluble entities do not undergo self-assembly into larger, readily detectable supramolecular structures in situ. In contrast, in eyes treated with FITC-Aβ42 fibrils, visualization of discrete positive signal was expected, provided the characteristic size and morphology of the assemblies. Indeed, in retinal sections obtained from these eyes, dispersed clusters of punctate fluorescence were identified at discrete regions along the inner retinal layers. The multifocal distribution of the highlighted structures that would suggest specific miniscule foci of damage contrasted with the general compromised ERG measures obtained in response to fibrillar Aβ42, which indicated a diffuse retinal dysfunction ensuing mainly from compromise of the photoreceptors and post-photoreceptoral neurons. Thus, we found no direct evidence for an immediate proximity of the amyloid fibrils and the primary site of retinal dysfunction which they mediate. It is thus speculated that a shift between the supramolecular architectures is possible under physiological conditions, and that a transition from fibrillar to oligomeric species could account for the mechanism of the toxicity occurring *in-vivo*. Accordingly, the extracellular Aβ deposits in drusen can potentially serve as a reservoir from which toxic species are being released to adjacent retinal cells, thus playing an important role in the disease mechanism^56–58^. Such suggested mechanism may account also for the experimental observations regrading Aβ40. In the case of this peptide, there was no evidence for the occurrence of early intermediates in the preparation, and consistently the fibrils were not toxic *in-vivo,* reflecting their apparent inability to allow transition into oligomeric species following the injection procedure.

The significance of Aβ-derived Müller cell reactivation, noted in this study and by others, and its consequences in the retinal physiology merit further investigation. While Müller cell activation in response to pathogenic Aβ species may represent a defense reaction as activated Müller cells are capable of clearing and degrading toxic Aβ species from the retina^35^, it may also disrupt the retinal homeostasis and therefore play a secondary role in the amyloid-mediated neurotoxicity. Presently, the contribution of Müller glial cells to the pathogenesis of Aβ-related retinopathy is yet to be fully understood.

Altogether, our results provide conceptual evidence of the *in-vivo* pathogenicity of Aβ in the retina, and promote the mechanistic understanding of the impact of particular Aβ species on the retinal integrity. Future studies to deepen the understanding of the long-term implications mediated by toxic supramolecular Aβ species on retinal cell degeneration may enhance our understanding of the fundamental pathophysiology of Aβ-related retinal degeneration, and may merit further investigation as a potential strategy for the treatment of AMD.

## Methods

### Preparation of Aβ assemblies

To ensure the initial monomeric state, 250 µg of commercially available Aβ40, Aβ42, or Fluorescein isothiocyanate (FITC) conjugated-Aβ42 (Bachem, Heidelberg, Germany) was dissolved in 250 µl of high-grade 1,1,1,3,3,3-hexafluoroisopropanol (HFIP) (Sigma-Aldrich, 105,228) by sonication for 20 s on ice and then by constant shaking at 150 rpm at 37 °C for 90 min. The samples were then aliquoted and the solvent was evaporated under high vacuum before storage at -20 °C until use.

Oligomers were produced according to Barghorn et al.^37^ Aliquots of the monomeric Aβ, normally 75 µg, were dissolved in 3.3 µl of dimethyl sulfoxide (DMSO) (Sigma-Aldrich) by sonication, before 33.9 µl of phosphate-buffered saline (PBS, pH 7.4) and 4.2 µl of a solution of 20% SDS were added. The solutions were aged at 37 °C without shaking for 6 h, at which point 82.8 µl of ultra-pure water (Biological industries, 03–055-1A) were added. The solutions were aged for additional 18 h at 37 °C.

In order to derive fibrilar assemblies, aliquots of 75 µg of the dry monomeric Aβ were dissolved in 16.1 µl of DMSO by sonication before 400 µl PBS were added. This mixture was aged at 37 °C without shaking for 24 h for Aβ42 fibrils formation, and 48 h for Aβ40 fibrils formation, respectively.

### ThT assay for fibril kinetics

50 µl of the fibril solution or blank, 50 µl of PBS and 10 µl of ThT solution (Sigma-Aldrich, T3516) (4 mM in PBS) were transferred together into black walled, clear- bottom 96 well plate and placed in the plate reader (Tecan Spark) at 37 °C, with a measurement (excitation at 450 nm, reading emission at 480 nm) taken every 10 min for 24–48 h.

### Gel electrophoresis

Utilizing a Tris-Tricine SDS-PAGE system the solutions were examined alongside a known protein marker (SeeBlue Pre-stained Protein Standard range 3–198 kDa, Invitrogen, LC5625), and stained with Imperial Protein Stain (Thermo Scientific, 24,615).

For the preparation of the SDS-PAGE system, gel buffer was prepared by dissolving 72.5 g Tris in 150 ml of DDW, the pH was brought to 8.5, then 3 ml of SDS 20% were added and the volume completed to 200 ml. Separating buffer was prepared by mixing 80 g of glycerol and 200 ml of gel buffer. The separating gel was prepared immediately before use by mixing 10 ml Acrylamide (30%, Bio-Rad, 1,610,154), 10 ml separating buffer, 66.25 µl ammonium persulfate (APS 10% in DDW, Sigma-Aldrich, A3678) and 6.6 µl N,N,N′,N′-Tetramethylethylenediamine (TEMD, Sigma-Aldrich, T9281). Then 0.75 mm gel stands were filled and covered with a layer of water which was dried with filter paper once the gel solidified. The stacking buffer was prepared by mixing 52.7 ml of gel buffer, 0.8 ml of SDS 20% and 140.3 ml of DDW. The Stacking gel was prepared immediately before use by mixing 1.25 ml Acrylamide, 5 ml stacking buffer, 50 µl of APS 10% and 5 µl TEMD. The top layer of the 0.75 mm gel stand was filled and an appropriate gel comb was inserted. The gel was left to solidify. Commercially available running buffer Tris/Tricine/SDS × 10 (Biorad, 1,610,744) (100 ml) was diluted into 900 ml of DDW. Of the standard ladder, 10 µl were run alongside 16 µl- samples of the Aβ solutions mixed with 6 µl of loading dye (Bio-Rad, 1,660,401). The system was run at 100 mV until the marker was spread out and the loading dye reached the lower portion of the gel. The gel was then removed from the system and stained using 10 ml of imperial protein stain (Thermo Scientific, 24,617) on a slow shaker for one hour, washed with water three times for 5 min each and then washed in water for an additional hour.

### Transition electron microscopy (TEM)

Samples (10 µl) were placed on copper grids (400 mesh) covered by carbon-stabilized Formvar film (EMS, BNFFA1000-Cu) After 2 min, excess fluid was removed, and the grids were negatively stained with 10 μl of 2% uranyl acetate solution for 2 min. Finally, excess fluid was removed, and the samples were viewed in a JEM 1400plus electron microscope operating at 80 kV.

### Retinal studies in animal model

All animals were treated in accordance with the ARVO statement for the Use of Animals in Ophthalmic and Vision Research and according to the Israeli Ministry of Health and institutional guidelines. The Ethics Committee of the Ruth Rappaport Faculty of Medicine, Technion, approved the study protocol. Adult Sprague–Dawley (SD) rats (male: female 1:1) were housed under 12/12-h light/dark cycles, with unrestricted access to food and water. Rats treated with FITC-Aβ assemblies were kept in dim light to prevent bright light exposure.

Each experimental group consisted of 9 rats, whereas 6 rats were included in each group treated with FITC-labelled Aβ assemblies. Prior to the intravitreal injection and electrophysiological tests, all rats were anesthetized with intramuscular injection (0.5 ml/kg) of a mixture consisting of Ketamine hydrochloride (10 mg/ml), Acepromazine maleate 10% and Xylazine 2% at a 10:2:3 proportion, respectively. Cyclopentolate hydrochloride 1% was used for full pupil dilation and topical Benoxinate HCl 0.4% was instilled for topical anesthesia. The rats were then injected with 10 µl of preformed Aβ assemblies solution to the vitreous chamber of the right (experimental) eye, whereas the left (control) eye was treated with the same volume of a blank solution containing only the vehicle. Briefly, a well-trained physician performed the injections using a standard 32-gauge needle inserted 1 mm posterior to the limbus, while employing indirect ophthalmoscopy to assure proper localization of the needle in the vitreous cavity. To avoid backflow and minimize dosage loss, the needle was stably held in the vitreous chamber for 2–3 s after the injection, before being slowly and steadily removed. Microsurgical forceps were used to gently seal the injection site for additional several seconds. Indirect ophthalmoscopy was repeated following the injection to rule-out unintentional damage to the retina or the lens from the procedure. ERG was performed at baseline, as well as 7 and 30 days after injection. At each time point following treatment, rats were sacrificed and the retinas were prepared for morphological assessment.

### Electroretinography (ERG)

Rats were dark-adapted overnight prior to ERG recording. Preparation of the ERG recording was conducted under dim red illumination. A heating pad was used to maintain each rat’s body temperature in the normal range. The ERG responses were recorded simultaneously from the experimental and control eyes as previously described^59^. Briefly, the system configuration consisted of corneal electrodes (LKC Technologies, USA) and stainless-steel reference and ground electrodes inserted into the ears in the dark-adapted state (UTAS-3000, LKC Technologies). The ERG responses were recorded by a custom-made software, using differential amplifiers (Grass Instrument Company, West Warwick, RI, USA), and a Ganzfeld light source (LKC Technologies, Gaithersburg, MD, USA) generating full field white light stimuli with a maximum strength of 5.76 cd-s/m^2^. The dark-adapted response consisted of six signals elicited by identical flashes applied at 10-s intervals.

For ERG analysis, the amplitude of the a-wave was measured from baseline to the trough of negative wave, and the b-wave amplitude was measured from the trough of the a-wave to the peak of the b-wave. The amplitudes of the dark-adapted b-wave of the experimental and control eyes of each rat were plotted as a function of the log flash strength. The response-stimulus strength relationships were fitted to a hyperbolic type function^60^ to derive their maximal amplitudes (Vmax). At each time point, the ratios between the maximal amplitudes of the ERG a-wave and b-wave obtained from the experimental and control eye in each rat were calculated. By comparing ERG measures from the experimental and control eyes, technical factors such as the depth of the anesthesia, body temperature or the duration of the dark-adaptation, which could possibly introduce inter-session variability of the ERG amplitudes and affect the assessment of retinal function, were thus minimized.

To quantitatively assess the change in the ERG, the relationship between the amplitude of the a-wave and the b-wave was determined for each response from each eye. The dependence of the b-wave on the a-wave was used as an index for the functional integrity of the photoreceptors, the ON-center bipolar cells, and signal transmission between them in the retina. The a-wave to b-wave amplitude ratio is expected to remain unchanged by disorders that affect only the photoreceptors, and to decrease when post-photoreceptoral elements are affected.

### Immunohistochemistry of rat retinal sections

The rats were sacrificed up to 5 weeks after injection. The eye was extracted and soaked for up to one hour in a solution of 2% paraformaldehyde and 2.5% glutaraldehyde in 0.1 M phosphate buffer (pH 7.4). For immunostaining, the eyecup was washed in 0.1 M PBS before being cryoprotected overnight in increasing concentrations of sucrose at 4ºC. The tissue was embedded in OCT and cut into 16-μm thick sections (Reichard Jung microtome). The cryostat sections were permeabilized in 1% TritonX-100 prior to blocking in 10% FBS (fetal bovine serum—02–023-5A-Biological industries), followed by overnight incubation in a moist chamber at 4 °C with a primary GFAP antibody (MAB3402 by Merck) diluted 1:2000 in 3% FBS + 0.1% TritonX-100. The secondary antibody (Alexa Fluor labelled donkey anti-mouse IgG, Thermo Fischer) was diluted 1:500 in 3% FBS + 0.1% TritonX-100. DAPI was added at 1:2000. As a control, sections from experimental eyes were stained with secondary antibody only. Samples were examined by a masked ophthalmologist using a Zeiss (Oberkochen, Germany) confocal LSM 700 microscope. For immunostaining analysis, the images were analyzed using Fiji software. Retinal areas were marked, and the fluorescence intensity was measured using the threshold tool.

### Histology of rat retinal sections

Rats were sacrificed up to 5 weeks after injection. The eyes were extracted and soaked in Hartman’s Fixative (Sigma- H0290) overnight. Eyes were processed through a graded series of EtOH, infiltrated, and embedded in JB4 (Electron Microscopy Sciences- 14,272–00). Ten micrometers sections were cut along the superior-inferior axis through the optic disc using a Leica microtome and stained with Methylene blue. Samples were examined using light microscope and the images were analysed using Fiji software. The software was programmed to recognize ONL and measure its area, and average ONL thickness was calculated as ONL area divided by retinal length.

## Supplementary information

Supplementary Information.
