# Supplementary material for: The retinal toxicity profile towards assemblies of Amyloid-β indicate the predominant pathophysiological activity of oligomeric species
Source: Sci Rep. 2020 Dec 1;10:20954. doi: 10.1038/s41598-020-77712-9 (PMC7708452; doi:10.1038/s41598-020-77712-9)
Supplement: Supplementary file 1 — Supplementary Information. [file 41598_2020_77712_MOESM1_ESM.pdf]

The Retinal Toxicity Profile towards Assemblies of Amyloid-β Indicate the Predominant Pathophysiological Activity of Oligomeric Species

Efrat Naaman [1§], Sarah Ya'ari [2§], Chen Itzkovich [3], Shadi Safuri [1,3], Flora Macsi [1], Lior Kellerman [4], Michael Mimouni [1,4], Irit Mann [4], Ehud Gazit [5], Lihi Adler-Abramovich [2*], Shiri Zayit-Soudry [1,3,4*]

(1) Department of Ophthalmology, Rambam Health Care Campus, Haifa 3109601, Israel

(2) Department of Oral Biology, The Goldschleger School of Dental Medicine, Sackler Faculty of Medicine, Tel Aviv University, Tel Aviv 69978, Israel

(3) Clinical Research Institute at Rambam, Rambam Health Care Campus, Haifa 3109601, Israel

(4) Ruth and Bruce Faculty of Medicine, Technion Israel Institute of Technology, Haifa, Israel

(5) School of Molecular Cell Biology and Biotechnology, Tel Aviv University, Tel Aviv 69978, Israel

Corresponding Authors
*E-mail: s_soudry@rambam.health.gov.il
*E-mail: lihia@tauex.tau.ac.il

§ Authors contributed equally

All authors have declared no conflict of interest.

Grant Support:
Israel Science Foundation, Physician-Scientist Grant Program 2346/16 (SZS)

A.

Aβ Oligomers

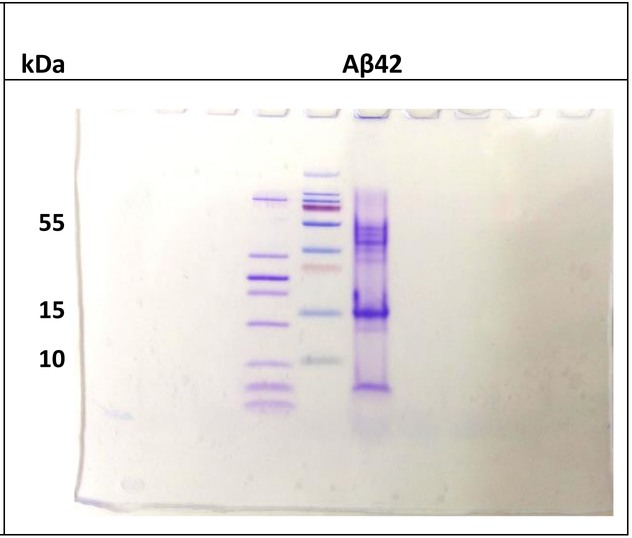

B.

Aβ Fibrills

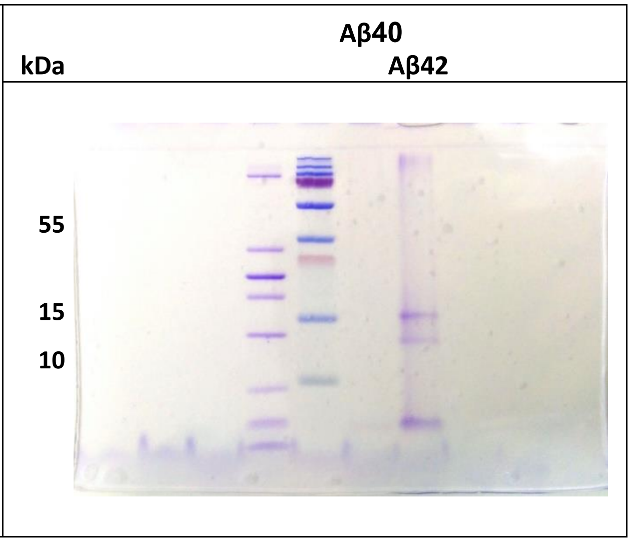

C.

Aβ FITC Oligomer (Oligo) And Fibrill (Fib)

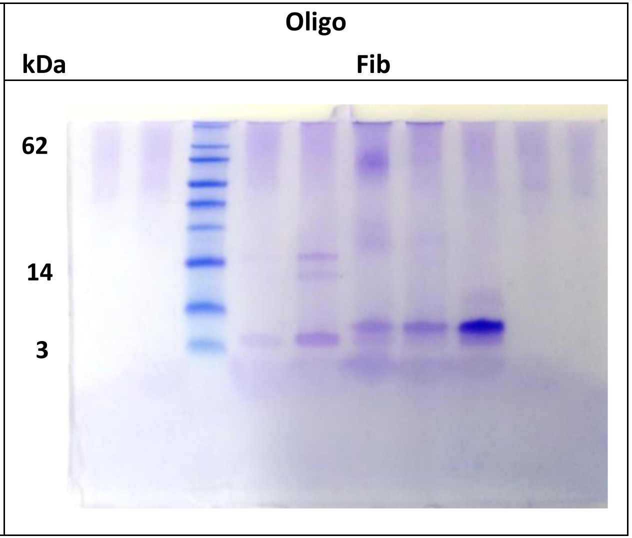

Figure S1. Full-length Gel electrophoresis for (A) oligomeric Aβ42, (B) fibrillar Aβ40 and Aβ42, and (C) oligomeric and fibrillar FITC-Aβ42 assemblies.

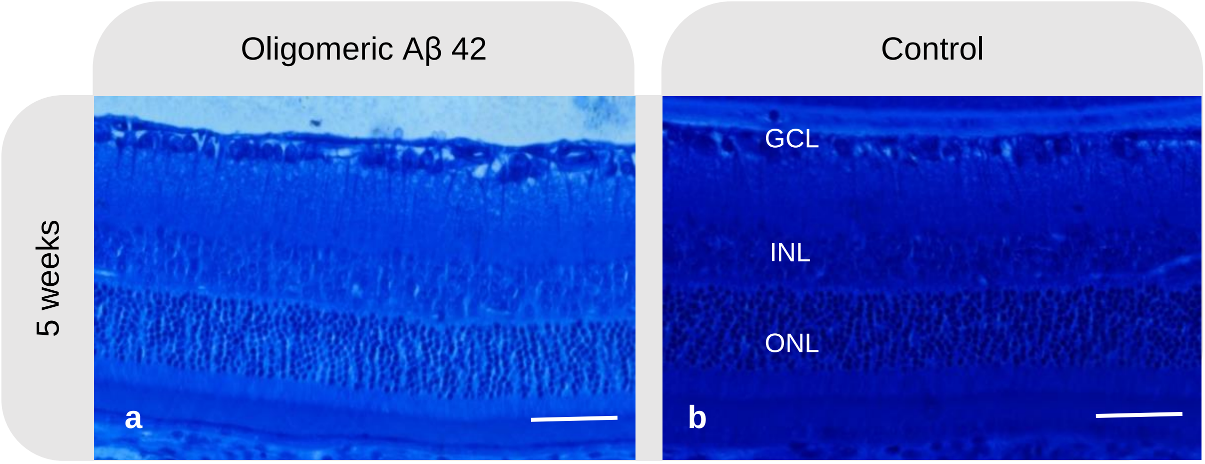

Figure S2. Histology of retina from a rat treated with intravitreal oligomeric Aβ42. Light photomicrographs of central retina stained with Methylene blue showing no differences in retinal layers' thickness between study (a) and control (b) eyes 5 weeks after intravitreal injection. Results were replicated in four rats in each subgroup. Scale bars: 50 µm.

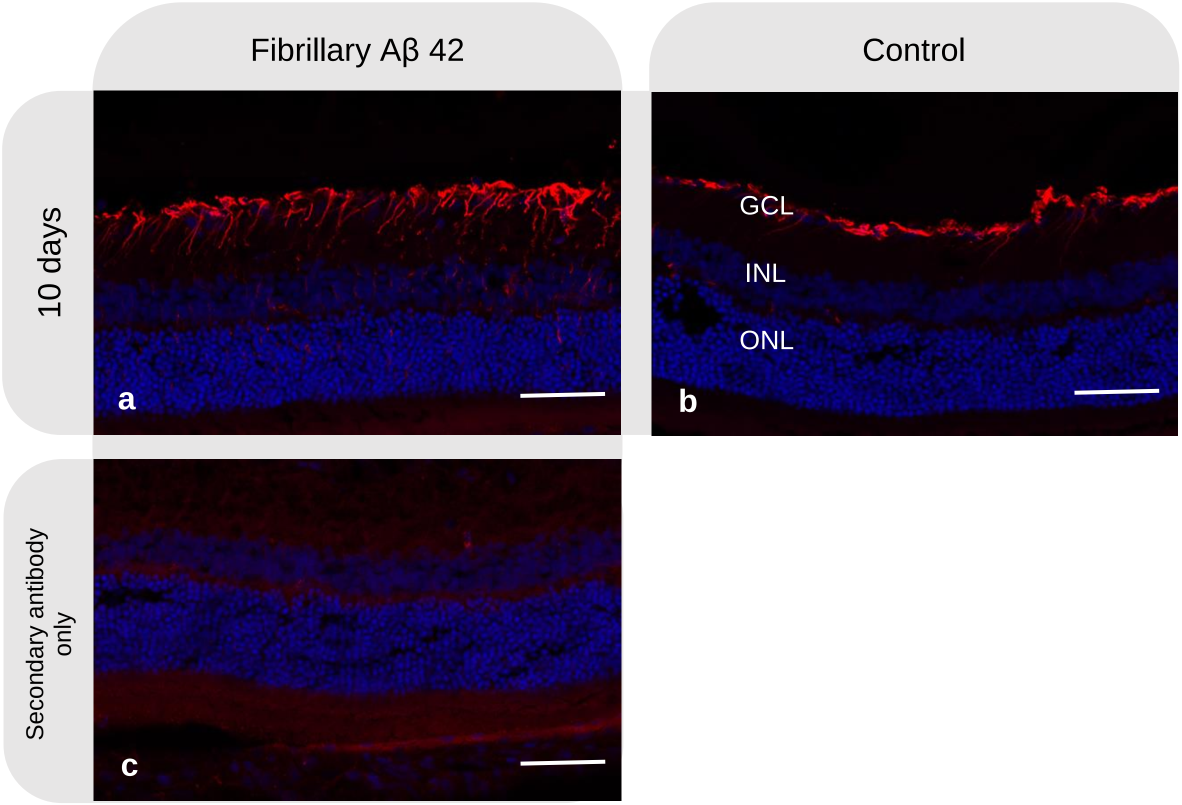

Figure S3. Immunostaining for glial fibrillary acidic protein (GFAP) in retinas of a rat treated with intravitreal fibrillary Aβ42. Retinal sections from the peripheral retina in the experimental eye (a) show significant GFAP staining of cells with a typical morphology for Müller cells. (b) Peripheral retinal areas from the control eye demonstrate no labelling of Müller cells. (Red- GFAP, Blue- DAPI staining of all nuclei). (c) As a control, sections from experimental eyes were stained with secondary antibody only. To obtain this data 16-µm tissue sections at five different tissue planes (100 µm apart) were prepared and stained. Results were replicated in four rats in each subgroup. Scale bars: 50 µm.
